# Endogenous non-retroviral elements in genomes of *Aedes* mosquitoes and vector competence

**DOI:** 10.1080/22221751.2019.1599302

**Published:** 2019-04-02

**Authors:** Vincent Houé, Mariangela Bonizzoni, Anna-Bella Failloux

**Affiliations:** a Department of Virology, Arboviruses and Insect Vectors, Institut Pasteur, Paris, France; b Collège Doctoral, Sorbonne Université, Paris, France; c Department of Biology and Biotechnology, University of Pavia, Pavia, Italy

**Keywords:** *Aedes albopictus*, arboviral diseases, vector competence, NIRVS

## Abstract

Recent extensive (re)emergences of arthropod-borne viruses (arboviruses) such as chikungunya (CHIKV), zika (ZIKV) and dengue (DENV) viruses highlight the role of the epidemic vectors, *Aedes aegypti* and *Aedes albopictus*, in their spreading. Differences of vector competence to arboviruses highlight different virus/vector interactions. While both are highly competent to transmit CHIKV (*Alphavirus,Togaviridae*), only *Ae. albopictus* is considered as a secondary vector for DENV (*Flavivirus, Flaviviridae*). Among other factors such as environmental temperature, mosquito antiviral immunity and microbiota, the presence of non-retroviral integrated RNA virus sequences (NIRVS) in both mosquito genomes may modulate the vector competence. Here we review the current knowledge on these elements, highlighting the mechanisms by which they are produced and endogenized into *Aedes* genomes. Additionally, we describe their involvement in antiviral immunity as a stimulator of the RNA interference pathways and in some rare cases, as producer of viral-interfering proteins. Finally, we mention NIRVS as a tool for understanding virus/vector co-evolution. The recent discovery of endogenized elements shows that virus/vector interactions are more dynamic than previously thought, and genetic markers such as NIRVS could be one of the potential targets to reduce arbovirus transmission.

The main vectors of many medically important arboviruses, such as chikungunya (CHIKV), zika (ZIKV) and dengue (DENV) viruses, are the two mosquito species *Aedes aegypti* and *Aedes albopictus.* While their extensive distribution covering most tropical, subtropical and even, temperate countries, makes them a real threat for human health, *Ae. aegypti* and *Ae. albopictus* have different historical backgrounds and do not exhibit the same efficiency to transmit arboviruses. The objectives of this review are to point out critical features of both mosquito species that could explain their differences in vector competence. Vector competence is modulated by environmental, genetic, and epigenetic factors, the latter including mechanisms induced by mosquito microbiota [1]. Recently, non-retroviral integrated RNA virus sequences (NIRVS) have been proposed to be among the genetic factors influencing vector competence. The potential role of NIRVS in mosquitoes as vectors is discussed.

## *Aedes albopictus* and *Aedes aegypti* have different histories (Figure 1)

*Aedes albopictus* (Skuse, 1894) is a mosquito species closely related to *Ae. aegypti*, both belonging to the Culicidae family and vectors of several different arboviruses highly pathogenic for humans such as chikungunya virus (CHIKV) [2,3], yellow fever virus (YFV) [4] and dengue viruses (DENV) [5,6]. Contrary to many other mosquito vectors such as the malaria vector *Anopheles gambiae*, *Ae. albopictus* and *Ae. aegypti* eggs are capable of entering in diapause and quiescence respectively, ensuring survival during and after environmental stress [7–10]. In addition to survive under extreme conditions, this characteristic allows the two vectors to colonize new regions around the world [11].

**Figure 1.**
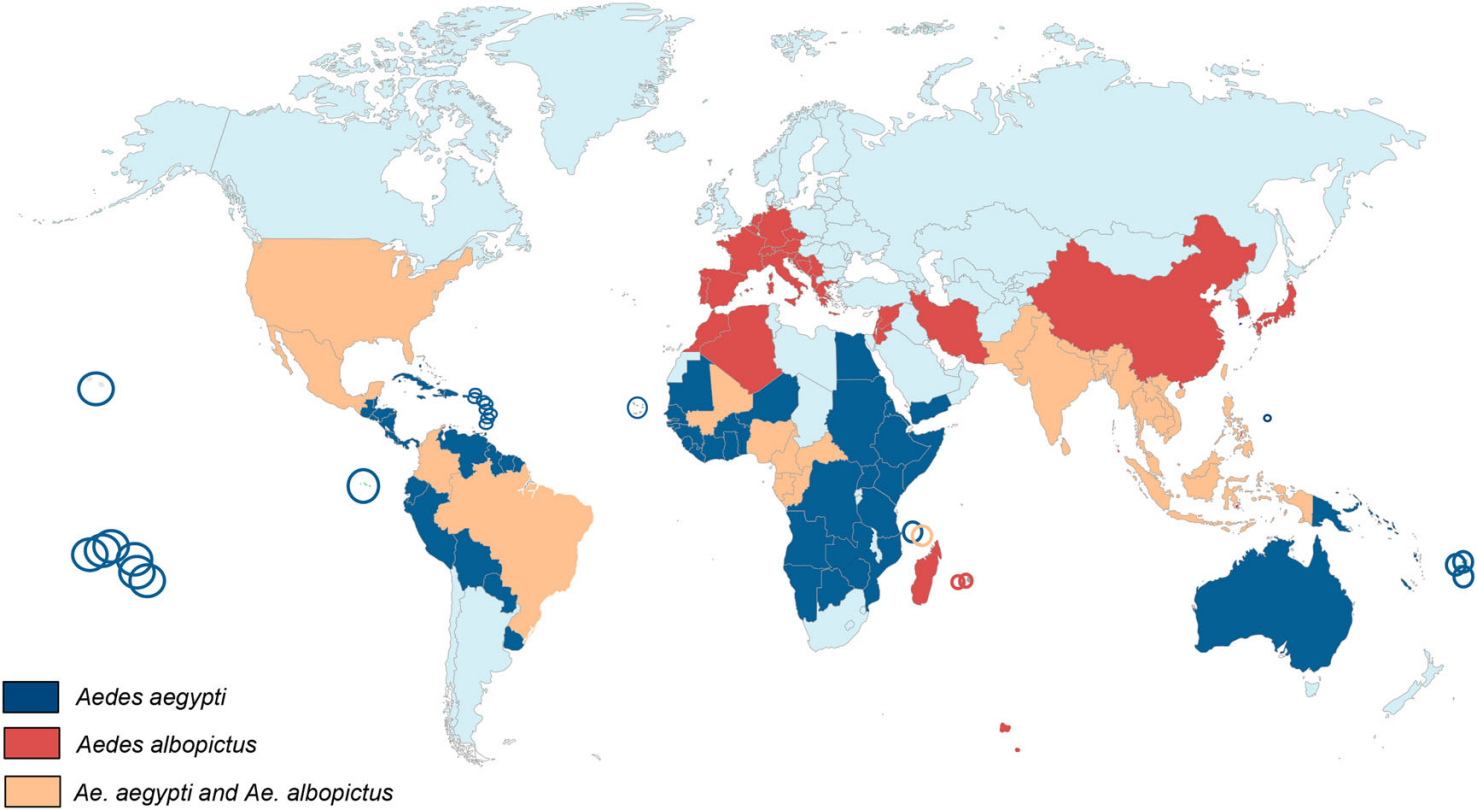
World distribution of *Aedes albopictus* and *Aedes aegypti*.

However, in terms of evolution, the two species have a different history. *Aedes aegypti* (Linné, 1862) originates from a sub-Saharan African sylvan ancestor that migrated to West Africa late in the 8th century. It was introduced in the New World probably via the African slave trade between 15th and 17th centuries [12,13]. Around 1800, the species was introduced in the Mediterranean region where it was established in European harbours until about 1950 [14]. *Aedes aegypti* was introduced into Asia from Europe with the opening of the Suez Canal in 1869; it is abundantly found in Asia since late nineteenth century [15]. The species was later introduced in Australia (1887) and the South Pacific (1904) [14]. On the other hand, *Ae. albopictus* is native to tropical forests of South-East Asia. Until the late 70s, this species was restricted to Asia, India and a few islands in the Pacific region such as La Reunion [16], the Seychelles [17] Mariana and Papua New Guinea islands [18]. However, in less than three decades, it has conquered all continents except Antarctica [19,20]. Contrary to *Ae. aegypti* which took hundreds of years to cover the tropical world, *Ae. albopictus* took only few decades to wide spread. This impressive fast colonization, promoted by increased human mobility and trade of goods including used tires and lucky bamboo as potential mosquito breeding sites, stresses its high ability to survive under both tropical and temperate regions. Moreover, *Ae. albopictus* is also a serious threat for human populations as it is a competent vector for at least 26 different arboviruses [21] and filarial nematodes of veterinary and zoonotic significance [22,23].

## Both species are involved as vectors in major human diseases (Table 1)

After suspected outbreaks in America and Asia in the 18th and 19th centuries [24], CHIKV has been first identified in Tanzania in 1952 where it circulated between non-human primates and mosquito vectors. The virus escaped from a sylvatic cycle to cause urban outbreaks in South East Asia and Africa from the 1960s with *Ae. aegypti* as the main vector (reviewed by [25]). Lastly, CHIKV re-emerged in Thailand in 1991 [26], in the Democratic Republic of Congo in 1999–2000 [27], then in coastal Kenya in 2004 [28], and in the Union of Comoros, in 2005 [29,30], mainly associated to *Ae. aegypti*. More recently, the same species was involved in CHIKV outbreaks in 45 countries and territories in America, causing almost 3 million cases from 2013 to 2016 (https://www.paho.org/hq/dmdocuments/2014/2014-jun-20-cha-CHIKV-authoch-imported-cases-ew-25.pdf; https://www.paho.org/hq/dmdocuments/2015/2015-sep-18-cha-CHIKV-cases-ew-37.pdf). On the other hand, *Ae. albopictus* was also proved to be susceptible to CHIKV infection [31] and could involve as a CHIKV vector. In 2005, it became the primary vector on La Réunion Island where *Ae. aegypti* was present as remote populations [32–34]. On this island, CHIKV acquired a mutation in the glycoprotein E1 (E1-A226V) [35] that increases its infectivity in *Ae. albopictus* but not in *Ae. aegypti* [36,37]. From there, the virus spread to India and Southeast Asia between 2007 and 2014 causing 1.4 million cases [38].

**Table 1.** List of arboviruses transmitted by Aedes aegypti and Aedes albopictu*s*.

Virus	Family	Genus	Transmitted by	
			*Aedes aegypti*	*Aedes albopictus*
DENV-1,2,3,4	*Flaviviridae*	*Flavivirus*	+	+
Yellow Fever Virus			+	+
West Nile Virus			+	+
Japanese Encephalitis Virus			-	+
St Louis Encephalitis Virus			-	+
Zika Virus			+	+
Usutu Virus			-	+
Chikungunya	*Togaviridae*	*Alphavirus*		+
Eastern Equine Encephalitis Virus			+	+
Venezuelan Equine Encephalitis Virus			+	+
Western Equine Encephalitis Virus			+	+
Ross River Virus			+	+
Sindbis Virus			+	+
Mayaro Virus			+	+
Getah Virus			+	+
Rift Valley Fever Virus	*Phenuiviridae*	*Phlebovirus*	+	+
Potosi Virus		*Bunyavirus*	-	+
Cache Valley Virus			-	+
Tensaw Virus			-	+
Keystone Virus			-	+
San Angelo Virus				+
La Crosse Virus			+	+
Jamestown Canyon Virus			-	+
Trivittatus Virus			-	+
Oropouche Virus			+	+
Orungo Virus	*Reoviridae*	*Orbivirus*	+	+
Nodamura virus	*Picornavirus*	*Nodaviridae*	+	+

*Aedes aegypti* and *Aedes albopictus* are also vectors of DENV, with the former being the major vector. *Aedes aegypti* has been responsible for severe outbreaks in America, Southeast Asia and Western Pacific regions in the late twentieth century [39,40]. On the other hand, even though it was also responsible for severe DENV outbreaks such as during the World War II in Japan [41] or recently in China [42], *Ae. albopictus* is considered as a less efficient vector of DENV. Indeed, no major epidemics were reported in regions like Taipei, Guam or Hawaii islands where *Ae. albopictus* is predominant, even when nearby places suffered from DENV outbreaks involving *Ae. aegypti* (reviewed by [43]). Additionally, even in presence of DENV outbreaks due to *Ae. albopictus*, like in the Seychelles Islands (1977), La Réunion Island (1977), southern China (1978), Macao (2001), Hawaii (2001) and lastly, in Europe [44,45], only mild symptoms are described [46].

## *Aedes albopictus* and *Aedes aegypti* have different vector competences

To be a vector, the arthropod species must be competent. The vector competence is the ability of an arthropod to acquire, support replication and dissemination of an infectious agent and successfully transmit it to another susceptible host [47,48]. Vector competence is a component of vectorial capacity and is determined by both genetic (depending on mosquito species/population, virus genotype/strain and their interactions) [49] and non-genetic factors (e.g. environmental components) [50]. *Aedes albopictus* and *Ae. aegypti* are highly susceptible to different CHIKV strains (Table S1) (reviewed by [51]). Although *Ae. albopictus* is considered as a secondary vector for DENV [21], its susceptibility to DENV infection compared to *Ae. aegypti* remains controversial [52–56]. *Aedes albopictus* mosquitoes generally show a higher midgut susceptibility to DENV infection but a lower rate of virus dissemination compared to *Ae. aegypti* (Table S2) [43].

Epigenetic factors which include mechanisms associated with the vector microbiota contribute to vector competence [1]. Insect microbiota comes from the environment: the breeding sites where immature stages live [57] and the flower nectar where adults get the sugar nutrient as carbon source [58]. Insect microbiota influences various physiological processes that favour insect ecological adaptation such as growth, reproduction, survival and tolerance to external stresses [59–63]. Moreover, insect microbiota is capable of stimulating immune responses, described as immune priming, conferring antiviral protection [64,65]. As an example, infection with the bacterium *Wolbachia* induces an oxidative stress in *Ae. aegypti* causing an increased level of reactive oxygen species (ROS). The elevation of ROS activates immune pathways, inhibit DENV and then affect the vector competence [66].

## Insect immunity: Toll, Imd, JAK-STAT pathways

The antiviral role of the microbiota has been ascribed to the activation of immune pathways [66,67]. So anti-viral immunity in mosquito vectors is critical to prevent virus replication and transmission. Mosquitoes lack adaptive immune responses, but they present innate immunity based on several strategies such as encapsulation and phagocytosis, melanization and production of physical barriers. Moreover, several molecular pathways have been described with antiviral immunity activities, including the RNA interference (RNAi) system, discussed further in the review, the Toll, Immune deficiency (Imd), Janus Kinase-Signal Transduction and Activators of Transcription (JAK-STAT) pathways [67–72]. The activation of these pathways leads to the expression of effector genes that have antiviral activities.

The Toll pathway of mosquitoes is very similar to the mammalian Toll-Like Receptor pathway (TLR). This pathway is activated by the interaction between either viral pathogen-associated molecular patterns (PAMPs) or the putative Toll ligand Spätzle, and host pattern recognition receptors (PRRs), that are present in several parts of the body (hemocele and midgut). This interaction leads to the recruitment of Myd88 that triggers the phosphorylation and degradation of the negative regulator Cactus and the nuclear translocation of the NF-kB-like transcription factor Rel1 that induces the transcription of antimicrobial peptides, such as cecropins and defensins [67].

The first (PRR activation) and the final step (synthesis of antimicrobial peptides) of the Imd pathway are processed in the same way as the Toll pathway, but different intermediate components are required in the cascade of the signalling pathway. The NF-kB-like transcription factor Rel2 is activated by the caspase-mediated cleavage and is then translocated to the nucleus where it triggers the transcription of Imd-related genes [73].

The JAK-STAT pathway is activated through the interaction between the Unpaired ligand (Upd) and the receptor Dome. It first promotes the binding of Janus kinases to Dome and then the recruitment of STAT proteins. Once activated, STAT proteins are translocated into the nucleus and trigger the transcription of antimicrobial related genes, and specific antiviral genes such as *vir-1* (virus-induced RNA 1) [74–76].

Studies in *Ae. aegypti* revealed that the Toll and JAK-STAT pathways were both upregulated 10 days after DENV infection suggesting an anti-DENV activity [67,77]. Moreover, the JAK-STAT pathway has an antiviral activity against another flavivirus, WNV in *Culex* mosquito cells [71]. However, although suggested in Drosophila [69,78], antiviral properties of these signalling pathways are less obvious for the alphaviruses of the Togaviridae family. In both *in vitro* and *in vivo* experiments with *Ae. aegypti* , none of the 3 above-mentioned pathways showed anti-CHIKV properties [79]. Additionally, in *Ae. albopictus*-derived U4.4 cells, infections with the *Alphavirus* Semliki Forest virus did not trigger the JAK/STAT, Toll and Imd pathways [80]. Primed by the mosquito microbiota, the Imd pathway showed antiviral effects in *Ae. aegypti* following a blood meal containing the *Alphavirus* Sindbis virus (SINV) [81]. Ultimately, a microarray analysis on *Ae. aegypti* infected by SINV revealed a temporary up-regulation of Toll pathway which was later inhibited by the virus [82]. Collectively, these results suggest that antiviral immunity in mosquitoes is in part controlled by the Toll, Imd and JAK-STAT pathways which are efficient against flaviviruses, such as DENV and WNV, but their action on alphaviruses such as CHIKV and SINV is less obvious suggesting a virus-specific antiviral regulation.

## Genome characteristics and evolution

### Quantitative trait loci (QTL)

Because vector competence is under the control of multiple genes, quantitative genetics have been used to measure the contribution of mosquito genetic factors to viral infection and dissemination in mosquitoes. Quantitative Trait Loci (QTL) are defined as several genes grouped in the genome that affect the expression of quantitative traits and lead to important phenotypic variations. The species *Ae. aegypti* is described under two forms: *Ae. aegypti formosus* for the ancestral African type breeding in tree holes and *Ae. aegypti aegypti* for the domestic type colonizing man-made containers [83]. Using intercrosses of *Ae. aegypti aegypti* and *Ae. aegypti formosus* strains, respectively highly and weakly susceptible to DENV infection, two QTLs were identified: one affecting the midgut infection barrier on chromosomes 2 and 3, and one on chromosome 3 associated with a midgut escape barrier [84]. Moreover, an additional QTL found on the chromosome 2 along with a sex-linked QTL were associated with the ability to infect the midgut [85]. Moreover, QTLs were identified on the 3 chromosomes of *Ae. aegypti* associated with DENV-2 dissemination from midguts [86]. It appears that several different parts of the *Ae. aegypti* genome identified as QTL are independently capable of modulating the vector competence to DENV-2. However, no studies to date have been conducted to identify potential QTLs affecting the vector competence to DENV in *Ae. albopictus* genome.

### Transposable elements (TE)

Last technical improvements in genome sequencing allowed bringing to light the complexity of mosquito genomes. *Aedes* mosquitoes have the biggest genome size among currently-sequenced mosquito genomes. For instance *Ae. aegypti* genome is 1,380 MB; [87], *Ae. albopictus* is 1,900 MB [88,89] while the *Anopheles gambiae* genome is 278 MB; [90] and *Culex quinquefasciatus* is 579 MB; [91].

Differences observed in the genome size of *Ae. albopictus* could be explained by the presence of Transposable Elements (TEs) [92,93]. First discovered in 1956 [94], TEs are considered as intragenomic parasites [95,96]. Ubiquitously found in both prokaryotic and eukaryotic genomes, TE are described as sequences integrated in the host genome capable of both independent replication and movement from one chromosomal location to another through a phenomenon called transposition. Transposition can occur in both somatic and germ line cells. However, some elements transpose in specific cell types, like the *P* elements in *Drosophila melanogaster* [97] or without any cells preference, such as the bacteriophage *Mu* [98,99]. Transposons are classified into two groups, depending on their DNA structure and transposition mechanism. The class I, also called retrotransposons, relies on RNA intermediates to transpose and is divided in two subgroups: LTR (Long Terminal Repeats) retrotransposons and non-LTR retrotransposons (reviewed by [100]). The class II TEs, also called DNA elements, contains terminal inverted repeats (TIRs). Three different groups of DNA elements have been described in eukaryotes: classic transposons [101], helitrons [102] and mavericks, also called politons [103]. Unlike retrotransposons, DNA elements do not rely on RNA intermediates for transposition [104].

Transposons are major drivers of host genome function and evolution. They can act as a source of mutational variations through their transposition producing multiple copies of the same element in the host genome. These copies can facilitate regulation of gene expression, recombination and unequal crossing-overs between chromosomes and therefore, lead to chromosomal rearrangements by creating deletions, insertions, duplications, inversions and translocations. When a TE insertion occurs in an exon, the ORF can change and codes for a non-functional peptide or cause missense or nonsense mutations. A TE insertion can also create alternative splicing leading to the production of several protein isoforms or introduce a polyadenylation signal [105,106]. TE activity in a host genome contributes to introduce diversity. In *Ae. albopictus* genome, the differences of genome size are explained by the amount of TEs which represents 68% (1,967 Mb) of the total genome [89]. Additionally, variations of repetitive sequences were detected at the intra- and interspecific levels [88,92,107,108]. When comparing the TE composition between *Ae. albopictus* and *Ae. aegypti*, differences in the quantity and type of repeats are seen; TE amount reaches 1,343 and 988 Mb in the *Ae. albopictus* and *Ae. aegypti* genomes, respectively [89]. More than 20% of repetitive sequences present in *Ae. albopictus* are absent in *Ae. aegypti*. The two species have diverged 71 million years ago and most TE insertions occurred during the last 10 million years in the *Ae. albopictus* genome [89]. DNA transposons represent only 8% of TEs present in the *Ae. albopictus* genome, and 15% in the *Ae. aegypti* genome [89]. Non-LTR retrotransposons LINE represent one third of TEs in both genomes, followed by a high proportion of LTR retrotransposons, suggesting that retrotransposons and DNA transposons are suspected to cause genome size variations between *Ae. aegypti* and *Ae. albopictus*. Moreover, the activity of TEs can be controlled by the siRNA and piRNA immune pathways. piRNAs and siRNAs produced respectively by TEs from class I and class II transposons, can be up-regulated after an infectious blood feeding leading to modify the outcome of infection, and then the vector competence [109].

### Endogenous viral elements (EVEs)

Due to strong and long-lasting interactions between the virus and the vector, the virus could integrate whole or parts of its genome into the genome of host cells, leading to the formation of Endogenous Viral Elements (EVEs) [110]. These elements are defined as viral sequences that integrate into the host germline as double-stranded DNA and are therefore maintained in the population through vertical transmission to the progeny. Considering that the genome of germline cells are strongly protected against any kind of intrusions, such as TE activity, notably by piRNAs [111], the odds of EVE introduction must be low. However, around 7%–8% of the human genome is made up by sequences of viral origins [112].

EVEs originated from retroviruses are called Endogenous Retroviruses (ERVs). It is well known that ERVs formation occurs frequently in host cells since the integration into the genome host cell is mandatory to complete their viral life cycle. ERVs are easily detectable because of Long Terminal Repeats (LTR) present at each end of the segment. Other EVEs originated from other viral families have been recently discovered in many host genomes: single-stranded DNA viruses such as *Circoviridae* and *Parvoviridae* in diverse vertebrate genomes (dog, mouse and panda; [113]) and double-stranded DNA viruses such as hepadnaviruses in zebra finch genome [114].

## Non-retroviral integrated RNA virus sequences (NIRVS) (Figure 2)

### Main characteristics

Since non-retroviral RNA viruses do not encode for reverse transcriptase or integrase, endogenous enzymes or viruses infecting the cell at the same time must be involved in the endogenization of such viruses into host genome DNA. Three steps should be involved to achieve the integration of non-retroviral RNA viruses into the host genome: (i) first, the non-retroviral RNA needs to be reverse-transcribed into viral-derived double-stranded DNA (vDNA), (ii) be imported in the nucleus, and (iii) finally be integrated into the host genome.

**Figure 2.**
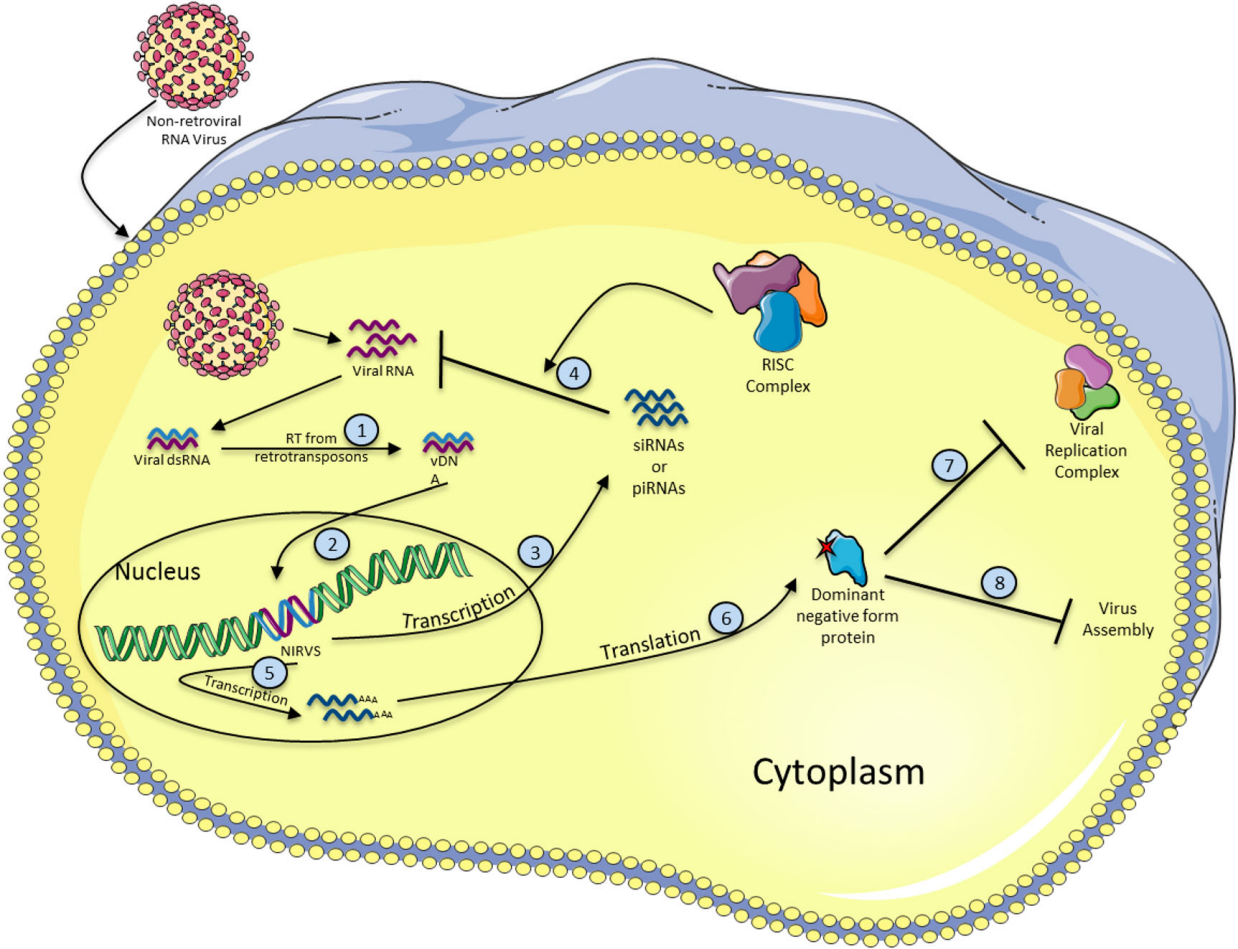
Formation and antiviral functions of NIRVS**.** When a non-retroviral virus infects a cell, the viral RNA is released and double stranded RNA (dsRNA) intermediates are produced. Viral dsRNA is then used as a template to produce viral DNA (vDNA) by the reverse transcriptase activity of retrotransposon elements (1). vDNA integrates into the host cell genome, probably by transposition activity of retrotransposons, becoming a NIRVS (2). NIRVS is then transcribed either into siRNAs or piRNAs (3) to inhibit the viral RNA after association with the RISC complex (4) or into mRNA (5), and translated into a dominant negative form protein (6), that can alter the viral replication by several ways. For example, by inhibiting the viral replication complex (7) or viral assembly (8).

The first mosquito NIRVS were identified in 2004 in *Aedes spp.* cell lines and mosquitoes [115]. Most of them were truncated or incorporated several stop codons, but one contained an intact ORF homologous to the NS1-NS4A region of insect-specific viruses (ISVs) in *Ae. albopictus* genome, i.e. Cell Fusing Agent Virus (CFAV) and Kamiti River Virus (KRV). This last fragment represents around one half of the flaviviral genome. These NIRVS (also called Cell Silent Agent (CSA) sequences) comprised two third of the flaviviral genome and contained enzymatic domains such as helicase and serine protease. The corresponding mRNA was detected in C6/36 *Ae. albopictus* cells suggesting the expression of the NIRVS and its potential functional role in the cell at the RNA level since no protein was detected [116]. Moreover, this NIRVS is present in 97%–98% of *Ae. albopictus* mosquitoes. Many NIRVS were found homologous to insect-specific flaviviruses (ISFs), such as CFAV, KRV and Aedes Flavivirus (AeFV) closely related to arboviruses [117,118]. The high prevalence of NIRVS in *Aedes spp*. genome as well as the high frequency of transposons might somehow be correlated to the mosquito genome size [87,89].

Most NIRVS described up to date were found in *Aedes* spp. genomes. Among 424 RNA viruses detected in 22 mosquito genomes, 81% (194/239) were identified as NIRVs in *Aedes* genomes and among them, 63% of NIRVS were located into the *Ae. aegypti* genome, and the remaining 37% were identified in *Ae. albopictus* [119]. Additionally, 72% of the NIRVS were homologous to the Rhabdovirus family whereas 27% were close to the *Flavivirus* genus and 1% left belonging to *Bunyavirus* and *Reovirus* genera. These data are consistent with another study which compared the “EVEome” of both *Ae. aegypti* and *Ae. albopictus* [120]. Factors leading to endogenisation of viral genomes into host cells remain unclear. The mRNA abundance could be critical since ssRNA+ genomes are directly translated into proteins and ssRNA- genomes have first to be transcribed. Moreover, the transcripts of flaviviral genome are usually longer than those from ssRNA- viruses, and this could decrease their chance to be integrated into the host genome [121]. Most of the flaviviral NIRVS detected *in silico* are originated from non-structural protein coding sequences rather than structural ones. In *Ae. aegypti* and *Ae. albopictus* genomes, 30 and 25 flaviviral NIRVS were mapped to non-structural protein coding sequences, and respectively, only 2 and 3 NIRVS represented similarities with structural proteins coding sequences [119]. Half of the rhabdoviral NIRVS mapped to the *N* gene, which encode the nucleoprotein [110,122]. From 3’ to 5’, each gene (N, P, M, G and L) of the rhabdoviral genome is transcribed in a progressive graduated manner due to the recognition of stop codons/polyadenylation signals by the polymerase [123], meaning that the transcripts at the 3’ end (i.e. N gene) are in higher quantities than for those near the 5’ end (i.e. L gene).

### Production of viral DNA (vDNA) from non-retroviral viruses

As previously mentioned, to become integrated into the host genome, the non-retroviral RNA virus is first reverse transcribed to produce viral DNA (vDNA), imported into the cell nucleus and finally integrate into the chromosome [124–127]. Interestingly, only some parts of the viral genomes can be found in a DNA form. The reverse transcriptase probably switches from the original RNA template to a close viral RNA genome causing multiple independent reverse-transcription events [128,129]. These vDNA could also be the result of replication-slippage events caused by the reverse transcriptase. Whether the vDNA form belongs to the host genome or is present as extra-chromosomal DNA element such as episomes is still unknown. RNAi-deficient cells (C6/36) possess more vDNA forms than RNAi-proficient cells (Aag2 cells) suggesting that RNAi system could inhibit vDNA production. More importantly, after mosquito infection with CHIKV, vDNA has been found in legs and wings of infected *Aedes* mosquitoes suggesting that either vDNA is capable of dissemination from one tissue to another (possibly through cellular and tissue damages) in the mosquito or that all infected cells produce vDNA [125]. Moreover, FHV and Sindbis vDNA were found in infected flies after infection [130].

### NIRVS reverse transcription and integration mediated by retrotransposons

vDNA from DNA viruses can integrate into host chromosomes by Non-Homologous (double-stranded) End Joining (NHEJ) [114,131], Non Homologous DNA recombination used by adeno-associated DNA virus [132–134] or Telomeric homologous recombination [135]. However, little is known about the mechanism used by the NIRVS to integrate into host chromosomes. Nevertheless, reverse transcription activity from endogenous retrotransposons has been associated with vDNA formation [130]. By adding a reverse transcriptase inhibitor, azidothymidine (AZT) in S2 and Kc167 *Drosophila* cell cultures, vDNA formation was inhibited after infection with several RNA viruses, namely Flock House Virus, Sindbis Virus and *Drosophila* C Virus (DCV) [130]. vDNA of CHIKV and DENV were detected after infections in *Ae. albopictus* and *Ae. aegypti* mosquitoes and cell cultures [125]. vDNA plays an important role in viral tolerance rather than viral resistance [125]. The early production of vDNA (6 hours and 2 days post-infection in cultured cells and mosquitoes respectively) is critical to establish efficient immune responses [125]. These regions were also enriched with LTR retrotransposons as it has been shown in *Drosophila*, especially retrotransposons of the Ty3_gypsy and Pao Bell families [119,120]. This suggests an important role of LTR retrotransposons in the reverse transcription of vDNA.

### Biological function of NIRVS

The integration of NIRVS into host genomes has now been recognized to occur more frequently than previously thought. It has been suggested that NIRVS could be involved in antiviral immunity [128,136]. A non-retroviral RNAs segment encoding the capsid protein of the Israeli Acute Paralysis Virus (IAPV), a ssRNA+ dicistrovirus, was found in the genome of one third of the honeybee population (*Apis mellifera*); it was correlated with a virus-resistant phenotype [137]. Moreover, the presence of vDNA allowed the survival of FHV-infected flies [125,130]. More precisely, vDNA production detected at early stages of infection, promoted viral persistence, as it has been seen in *in vivo* and *in vitro* experiments with mosquitoes challenged with CHIKV and S2 FHV-infected *Drosophila* cells [125,130].

The antiviral function of NIRVS has been linked to the innate immune system of RNAi which has been shown to be the main antiviral system in insects [125,138,139]. This system relies on small RNAs (sRNA) that when associated with a complex of proteins recognized by sequence-complementarity, led to the cleavage and degradation of incoming foreign nucleic acids [140]. Three different pathways have been described: the small interfering RNA (siRNA), the micro RNA (miRNA) and the PIWI-interacting RNA (piRNA). All three use the same mechanism to perform their antiviral action, but are distinguished by the sRNA biogenesis and the protein complex involved. Whereas the role of siRNA pathway in viral immunity in mosquitoes is largely accepted, little was known about the function of the piRNA pathway except its role in preserving genome stability in the germline by regulating the activity of transposable elements in *D. melanogaster* [141–145] and *Aedes* mosquitoes [146,147]. However, the piRNA pathway has been linked to antiviral immunity both *in vitro* and *in vivo* [148–153]. Indeed, deep-sequencing analysis of DENV-2 infected *Ae. aegypti* Aag2 cells revealed the production of specific viral piRNAs (vpiRNAs) along with viral siRNAs (vsiRNAs) [154]. Moreover, vpiRNAs have been detected in DENV-infected *Ae. aegypti* individuals as early as 2 days post-infection [151]. Nevertheless, the piRNA pathway has no antiviral property in the insect model *D. melanogaster* suggesting a different function depending on the host [155].

Interestingly, EVEs including NIRVS present in *Aedes* mosquitoes are frequently located in TE-derived piRNA clusters [119,120]. In *Ae. aegypti* and *Ae. albopictus,* half of NIRVS mapped to piRNA clusters in *Ae. aegypti* genome and only 12.5% of NIRVS mapped to piRNA clusters in *Ae. albopictus* genome [119], suggesting that the presence of NIRVS in these clusters was not a general feature. Moreover, bioinformatic predictions on Aag2 cell line showed that piRNA clusters containing EVEs produced more piRNA than those without EVEs, meaning that viruses may not integrate randomly in the host genome but target specific active piRNA clusters for endogenization [120]. Additionally, NIRVS produced both primary and secondary piRNAs; immunoprecipitation of Piwi proteins also detected NIRVS-derived sRNAs, and knockdown of Piwi proteins resulted in a decrease of NIRVS-derived sRNA expression [119]. However, NIRVS-derived siRNAs were not found indicating that NIRVS are involved in only one specific RNAi pathway. NIRVS originated from insect-specific viruses were proved to produce antisense orientation primary piRNA-like molecules and be located in active regions of both siRNA and piRNA production in *Ae. aegypti* and *Ae. albopictus* mosquitoes [116]. In CHIKV-infected *Ae. aegypti* and *Ae. albopictus*, NIRVS produced viral small-interfering RNAs (vsiRNAs) and probably vpiRNAs after infection [125]. In FHV-infected *Drosophila* cells treated and non-treated with AZT (inhibitor of reverse transcriptase), vDNA are transcribed and processed by the RNAi machinery into vsiRNAs [130]. The knocking-down of RNAi machinery in *Drosophila* infected cells resulted in an acute infection leading to cell death [130].

In summary, NIRVS located in specific regions of the genome such as TE-derived areas called piRNA clusters in mosquitoes, are important for RNAi-based immunity [156]. Their transcripts are capable of producing vsiRNAs in *Drosophila* [130] and both vsiRNAs and vpiRNAs in *Aedes* mosquitoes [119,120,125]. The production of sRNAs is induced following arboviral infections (Togaviridae and Flaviviridae) and NIRVS are required for mosquito tolerance to control viral infection [125]. Since vDNA has been found in many mosquito tissues following viral infection, vDNA could serve as a danger signal to warn the uninfected cells and implement a solid immune response through sRNA production [125], even though the virus could also counteract by producing VSR (Viral Suppressor of RNAi), as it has been seen with insect-specific viruses [157,158].

### NIRVS functional role at the protein level

Even though some NIRVS have accumulated several mutations including stop codons, some of them have conserved their open reading frames (ORFs) suggesting that they could be translated into proteins and have a function at the protein level. This scenario was first described for many Endogenous Retroviral elements (ERVs) found in different host genomes [159]. Produced proteins can confer viral interference and direct antiviral properties, leading to a resistance phenotype [160–162]. Up to now, no biological functions were found at the protein level for NIRVS in mosquitoes. However, many of them were proved to produce transcripts, mostly in *Aedes* and *Anopheles* mosquitoes [115,116,119,122,163] meaning that related proteins should be discovered shortly. Collectively, these results suggest that NIRVS have biological functions rather than being endogenized randomly into host genomes. Despite their low or even undetectable levels of RNA [119], NIRVS are suggested to be involved in the main antiviral defense mechanism in mosquitoes as being a source of sRNA production [116,119,120,125,130]. In some rare occasions, NIRVs produce a protein which blocks viral infection and replication by affecting viral polymerase activity [161].

### NIRVS as ancient scars attesting virus/host coevolution

Understanding ancient viral cross-species transmission events and how viruses have evolved and interacted with their hosts in the past is important for anticipating future emerging diseases. However, reconstituting the history of viruses remains a challenge considering their rapid evolution. Indeed, viruses are considered as the fastest-evolving biological entity with an evolution rate of 10^−3^ substitutions/site/year (s/s/y) [164–167]. Once endogenized in the host, NIRVS are submitted to a slower evolution rate, around 10^−9^ s/s/y for mammals [165,168]. However the evolutionary reconstruction of the NIRVs remains tricky. EVEs are considered as « fossil records » of ancient infections [169]. Several different methods have been described to date EVEs [110,170]. The minimum insertion date of the EVE can be evaluated if the divergence time of the two species sharing the same taxonomic position is known [110]. Studies on EVE evolution revealed that many viral families are more ancient than previously thought. As an example, the lentivirus family classified as retroviruses dated to a hundred years by molecular clock dating techniques [171] appeared several million years ago since endogenous lentiviruses were discovered in the grey mouse lemur (*Microcebus murinus*) from Madagascar [172]. This can be extended to other viruses: *Hepadnaviridae* [114,173] and *Bornaviridae* [110].

## Conclusion

*Aedes albopictus* and *Ae. aegypti* are two mosquito species that have different histories. They vector several major human arboviruses, including CHIKV and DENV, for which they exhibit different vector competence. Whereas both species highly transmit CHIKV, *Ae. albopictus* is considered as a less efficient vector for DENV [21]. Along with environmental factors such as the temperature, epigenetic factors like the mosquito microbiota [1,66,67], and genetic factors like Quantitative Trait Loci (QTLs) [86] are important to determine the vector competence. More importantly, the recent discovery of NIRVS highlights their potential role as modulator of vector competence to arboviruses. It has been suggested that their association with retrotransposons allowed them to be reverse transcribed into viral DNA (vDNA) and then be integrated into mosquito genomes. Moreover, NIRVS were found to produce vsiRNAs and vpiRNAs, which are important molecules in the RNAi-based immunity in *Aedes* mosquitoes [119,120,125]. In rare cases, NIRVS are translated into proteins that act as inhibitor of viral replication [161]. However, some questions remain unsolved, such as to which aim NIRVS are involved in the antiviral immunity. NIRVS could act as a warning signal and prime the antiviral immunity for allowing the host to control viral replication before the infection becomes deleterious and harmful for the vector host. It reminds us the adaptive immunity mechanisms such as CRISPR-Cas systems in prokaryotic cells. Alternatively, NIRVS could also act as a keeper of persistent infection by maintaining a low level of viral replication, diminishing the negative impacts on the mosquito fitness. Nevertheless, analysis of natural mosquito populations revealed a high diversity of NIRVS at the intra- and inter-population levels (Houé et al. unpublished data; [174]), suggesting many DNA recombination in NIRVS-surrounding areas. While many NIRVS have been found homologous to insect-specific flavi- and rhabdovirus [119], which are genetically related to pathogenic viruses, none was found homologous to Togaviridae family that contains only two insect-specific viruses described so far [175,176]. This could explain why CHIKV is highly transmitted by both *Ae. aegypti* and *Ae. albopictus*, compared to the Flaviviridae family that harbours many insect-specific viruses [177].

## Supplementary Material

Supplemental Material
