# Supplementary material for: Endogenous non-retroviral elements in genomes of Aedes mosquitoes and vector competence
Source: Emerg Microbes Infect. 2019 Apr 2;8(1):542–55. doi: 10.1080/22221751.2019.1599302 (PMC6455143; doi:10.1080/22221751.2019.1599302)
Supplement: Supplemental Material [file TEMI_A_1599302_SM9139.docx]

**Supplementary information**

Supplementary information accompanies the manuscript on the *Emerging Microbes & Infections* website http://www.nature.com/emi
